# Targeting the insulin-like growth factor-1 receptor in human cancer

**DOI:** 10.3389/fphar.2013.00030

**Published:** 2013-03-22

**Authors:** Alexandre Arcaro

**Affiliations:** Division of Pediatric Hematology/Oncology, Department of Clinical Research, University of BernBern, Switzerland

**Keywords:** cancer, clinical trials, insulin-like growth factor, IGF-1 receptor, monoclonal antibody, tyrosine kinase inhibitor

## Abstract

The insulin-like growth factor (IGF) signaling system plays a crucial role in human cancer and the IGF-1 receptor (IGF-1R) is an attractive drug target against which a variety of novel anti-tumor agents are being developed. Deregulation of the IGF signaling pathway frequently occurs in human cancer and involves the establishment of autocrine loops comprising IGF-1 or IGF-2 and/or IGF-1R over-expression. Epidemiologic studies have documented a link between elevated IGF levels and the development of solid tumors, such as breast, colon, and prostate cancer. Anti-cancer strategies targeting the IGF signaling system involve two main approaches, namely neutralizing antibodies and small molecule inhibitors of the IGF-1R kinase activity. There are numerous reports describing anti-tumor activity of these agents in pre-clinical models of major human cancers. In addition, multiple clinical trials have started to evaluate the safety and efficacy of selected IGF-1R inhibitors, in combination with standard chemotherapeutic regimens or other targeted agents in cancer patients. In this mini review, I will discuss the role of the IGF signaling system in human cancer and the main strategies which have been so far evaluated to target the IGF-1R.

## THE IGF/INSULIN FAMILY OF GROWTH FACTORS

The insulin-like growth factor (IGF)/insulin family of growth factors is an evolutionally conserved system which plays a crucial role in the growth and development of many tissues and the regulation of overall growth and metabolism. This system comprises three receptors [insulin receptor (IR), IGF-1 receptor (IGF-1R), and IGF-2/mannose 6-phosphate receptor (M-6-PR)], three ligands (insulin, IGF-1, and IGF-2), and six known types of circulating IGF-binding proteins (IGFBP1–6; Pollak et al., 2004; Pollak, 2008). The IGF-1R is a receptor tyrosine kinase which is widely expressed in many human tissues and cell types and is highly homologous to the IR. However, these two receptors have distinct functions, since the IGF-1R controls apoptosis, cell growth, and differentiation, while the IR regulates physiological processes. The IGF-1R is a heterotetrameric glycoprotein composed of two α and two β subunits, post-transcriptionally linked by disulfide bonds. Activation of the IGF-1R is achieved by binding of its specific ligand to the extracellular α subunits, which leads to autophosphorylation of three tyrosine residues within the kinase domain of the IGF-1R β subunit.

Insulin-like growth factors 1 and 2 are single-chain polypeptides with a high sequence homology to pro-insulin. The half-lives, transportation, and bioavailability of the IGFs circulating at high concentrations in the bloodstream and extracellular fluids are modulated by several high affinity IGF-binding proteins (IGFBP1–6). More than 99% of the circulating IGFs are bound to IGFBPs and the IGFBPs themselves are tightly regulated by tissue specificity, cell or matrix association, phosphorylation, and proteolysis by various proteases (Baxter, 2000).

Both IGF-1 and IGF-2 interact with the IGF-1R, although IGF-1 shows a much higher affinity than IGF-2. The IGF-2 receptor (M-6-PR), differs significantly from the IGF-1R and does not activate specific cellular responses. The ability of the highly homologous IGF-1R and IR to form hybrid receptors by dimerization further increases the complexity of the signaling system. Such IGF-1R/IR hybrid receptors have been reported to influence cell responses by altering the affinities of their growth factor ligands (Pandini et al., 2002; Pollak, 2008; Gallagher and LeRoith, 2010). These hybrid receptors can stimulate cell proliferation, especially in the case of the IR-A isoform, which has been found over-expressed in cancer (Denley et al., 2003).

**FIGURE 1:**
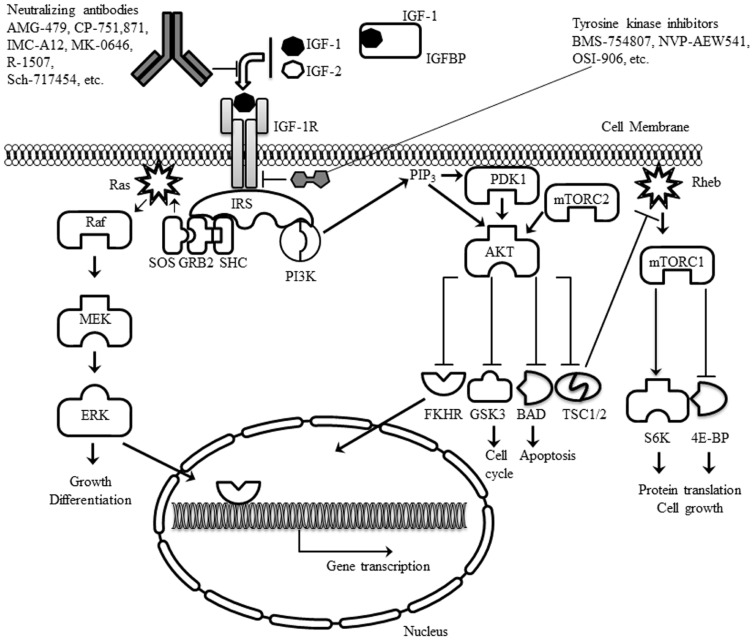
**Schematic representation of the IGF-1R, its ligands and the main intracellular signaling pathways activated.** The two main classes of experimental drugs against the receptor (antibodies and tyrosine kinase inhibitors) are also depicted.

The main intracellular signaling pathways downstream of the IGF-1R use the IR substrates-1 to -4 (IRS-1 to -4) and the Src-homology collagen protein (Shc) isoforms as adapter molecules. Phosphorylation of the IRS adapter molecules on one hand triggers activation of the phosphoinositide 3-kinase (PI3K)/Akt signaling pathway, whereas, on the other hand, the Shc adapter activates signaling by the Ras/Raf/MEK/Erk signaling pathway (**Figure 1**). Generally, signals controlled by the IGF-1R have pleiotropic effects on cell behavior controlling cell proliferation, differentiation, and cell migration, but also regulating the apoptotic machinery (Pollak, 2008; Gallagher and LeRoith, 2010).

## THE ROLE OF THE IGF-1R IN HUMAN CANCER

In the past decades, a large body of evidence has arisen, supporting a key role for IGF-1R signaling in various types of human cancers (Pollak, 2008; Gallagher and LeRoith, 2010). A number of studies performed in the last two decades have demonstrated a role for this receptor in the transformation of cells, cancer cell proliferation, as well as in metastatic events (Kaleko et al., 1990; Sell et al., 1994; Scotlandi et al., 2002; Sachdev et al., 2004; Carboni et al., 2005).

While no recurrent cancer-specific mutations of the IGF-1R or its ligands have been described to date, a plethora of studies have provided evidence for a link between this signaling pathway and the risk of developing cancer (Khandwala et al., 2000; Pollak, 2008; Gallagher and LeRoith, 2010). The most common findings associated with deregulated IGF signaling are over-expression of the IGF-1R or the establishment of autocrine or paracrine signaling loops. While high expression levels of the IGF-1R have been found in breast and colorectal cancer, autocrine signaling loops are more common, and have been reported in a wide variety of human malignancies. Paracrine signaling has mainly been described for breast cancer, where stromal cells have been shown to produce IGF-1 and IGF-2. Population studies have further highlighted the importance of IGF signaling in some of the most common cancers (Guerreiro et al., 2006a; Pollak, 2008; Gallagher and LeRoith, 2010). The published evidence from epidemiological studies has revealed a correlation between elevated IGF-1 levels and an increased risk of cancer diagnosis (Pollak et al., 2004; Guerreiro et al., 2006a; Pollak, 2008; Gallagher and LeRoith, 2010). Although the population studies did not always come to the same conclusions, systematic reviews of these results led to the interpretation that circulating IGF-1 levels are indeed related to a risk of several common cancers (Renehan et al., 2004). The most significant correlation between increased levels of IGF-1 and the risk of cancer diagnosis was found for prostate cancer, pre-menopausal breast cancer, and colorectal cancer (Wolk et al., 1998; Ma et al., 1999; Giovannucci et al., 2000; Harman et al., 2000; Kaaks et al., 2000; Stattin et al., 2000; Chan et al., 2002; Palmqvist et al., 2002; Chen et al., 2009; Major et al., 2010; Rinaldi et al., 2010). However, it should be noted that no significant overall associations were found between breast cancer and common germline variation in *IGF1* and other genes involved in IGF-1 metabolism in a large, comprehensive study (Canzian et al., 2010).

In summary, mechanistic and epidemiological studies have provided substantial information supporting a role for IGF signaling and the IGF-1R in human cancers. The IGF-1R has emerged as a promising target for the development of new therapeutic approaches, which can be combined with other classical treatment regimens.

## STRATEGIES TO TARGET THE IGF-1R IN CANCER THERAPY

The IGF-1R can be inhibited through various experimental approaches (**Figure 1**). I will focus the discussion on the two approaches which are currently being evaluated in clinical trials: (A) neutralizing antibodies and (B) small molecule inhibitors of the IGF-1R tyrosine kinase activity.

## NEUTRALIZING ANTIBODIES

A number of monoclonal antibodies have been developed to target the receptor itself, which bind to the extracellular domains of the IGF-1R and block ligand binding. A feature common to all anti-IGF-1R antibodies, probably more important than the blocking activity itself, is their ability to down-regulate of the IGF-1R overtime by promoting internalization of the receptor. Receptor-targeting antibodies might have important therapeutic advantages, concerning both specificity and toxicity. A variety of fully human anti-IGF-1R monoclonal antibodies have been characterized and showed strong anti-tumor activity *in vitro* and *in vivo *(King and Wong, 2012). Most IGF-1R antibodies which have been evaluated in clinical trials so far have proven to be well tolerated (King and Wong, 2012). A selection of the results published with these molecules is described below.

AMG-479 (ganitumab; Amgen) is a fully human immunoglobulin G1 (IgG1) against the IGF-1R (Beltran et al., 2009). AMG-479 blocks IGF-1 and IGF-2 binding to the IGF-1R without cross-reacting with the IR and also inhibits the activation of IGF-1R homodimers and IGF-1R/IR hybrids (Beltran et al., 2009). A phase I study in patients with advanced solid malignancies or non-Hodgkin’s lymphoma showed that AMG 479 can be administered safely and tumor responses were observed in patients with Ewing/primitive neuroectodermal tumors and neuroendocrine tumors (Tolcher et al., 2009). The efficacy and safety of ganitumab combined with gemcitabine was investigated in a randomized phase II trial in patients with metastatic pancreatic cancer (Kindler et al., 2012). The combination had tolerable toxicity and showed trends toward improved survival rate and overall survival (Kindler et al., 2012). Another phase II study with ganitumab as a monotherapy in patients with metastatic Ewing family tumors or desmoplastic small round cell tumors showed that it was well tolerated and demonstrated activity in both tumor types (Tap et al., 2012). Several other phase II clinical trials evaluating ganitumab, alone and in combination with other anti-cancer agents, are ongoing in patients with various types of solid tumors.

CP-751,871 (figitumumab; Pfizer) is a fully human IgG2 antibody which blocks binding of IGF-1 to its receptor, IGF-1-induced receptor autophosphorylation and induces the down-regulation of IGF-1R (Cohen et al., 2005; Gualberto, 2010). A phase I study in patients with refractory solid tumors showed that figitumumab has a favorable safety profile and is well tolerated (Haluska et al., 2007). A phase I study in patients with multiple myeloma confirmed the favorable profile of this agent and some responses were reported in patients treated with figitumumab in combination with dexamethasone (Lacy et al., 2008). Another phase I study in patients with sarcoma and Ewing’s sarcoma found figitumumab to be well tolerated and had anti-tumor activity in Ewing’s sarcoma (Olmos et al., 2010). A phase Ib study tested figitumumab in combination with docetaxel in patients with advanced solid tumors and found this regimen to be well tolerated (Molife et al., 2010). A phase I trial of the combination of everolimus and figitumumab was conducted in patients with advanced sarcomas and other solid tumors (Quek et al., 2011). The combination appeared safe and exhibited interesting anti-tumor activity warranting further investigation (Quek et al., 2011). In squamous cell carcinoma of the head and neck, figitumumab showed no activity in a phase II trial as a single agent (Schmitz et al., 2012). Although figitumumab induced a down-regulation of the IGF-1R, an activation of the epidermal growth factor receptor (EGFR) pathway was noted (Schmitz et al., 2012), which may contribute to resistance to this agent. Despite these results, two large-phase III trials investigating the addition of figitumumab to either carboplatin/paclitaxel (NCT00596830), or to erlotinib (NCT00673049) in advanced non-small cell lung cancer (NSCLC) patients were terminated after planned interim analysis indicated futility.

IMC-A12 (cixutumumab; ImClone Systems Incorporated) is a fully human monoclonal anti-IGF-1R IgG1 antibody, which inhibits receptor activation, downstream signaling and also mediates internalization and degradation of the receptor (Rowinsky et al., 2007). Although promising single-agent activity was observed, the most impressive effects of targeting the IGF-1R with IMC-A12 were observed when this agent was combined with cytotoxic agents or other targeted therapeutics (Rowinsky et al., 2007). The results of a phase II study of IMC-A12, with or without cetuximab, in patients with refractory metastatic colorectal cancer documented that IMC-A12 alone, or in combination with cetuximab, was insufficient to warrant additional study in patients with colorectal cancer refractory to EGFR inhibitors (Reidy et al., 2010). IMC-A12 was evaluated in combination with the mammalian target of rapamycin (mTOR) inhibitor temsirolimus in patients with refractory Ewing’s sarcoma family tumors (Naing et al., 2012). The combination was well tolerated and showed preliminary evidence of durable anti-tumor activity (Naing et al., 2012). Currently several clinical trials are evaluating IMC-A12 as a single agent or in combination with standard chemotherapy or other targeted agents.

MK-0646 (dalotuzumab, h7C10; Merck) is a humanized IgG1 monoclonal antibody against the IGF-1R (Goetsch et al., 2005; Scartozzi et al., 2010). Pre-clinical studies have demonstrated that dalotuzumab acts by inhibiting IGF-1- and IGF-2-mediated tumor cell proliferation (Goetsch et al., 2005), IGF-1R autophosphorylation, and Akt phosphorylation (Wan et al., 2007; Cao et al., 2008; Broussas et al., 2009; Scartozzi et al., 2010). Data from phase I clinical trials demonstrated that dalotuzumab is safe, well tolerated and significantly inhibits tumor proliferation (Scartozzi et al., 2010; Atzori et al., 2011). A phase II study evaluated the safety and efficacy of MK-0646, as monotherapy in patients with metastatic, well-differentiated neuroendocrine tumors (Reidy-Lagunes et al., 2012). MK-0646 was inactive as a single agent and thus further studies of MK-0646 as a monotherapy in unselected neuroendocrine tumors are not warranted (Reidy-Lagunes et al., 2012). Several clinical trials evaluating dalotuzumab, alone and in combination with other anti-cancer agents, are ongoing in patients with various types of solid tumors and in patients with multiple myeloma.

R-1507 (robatumumab, Roche) is a fully humanized anti-IGF-1R monoclonal antibody. In a panel of NSCLC cell lines predictive biomarkers of response to R-1507 were investigated. While levels of phospho-IGF-1R did not correlate with drug sensitivity, the sensitive NSCLC cell lines displayed high levels of total IGF-1R and higher copy numbers (Gong et al., 2009). Different studies reported on the enhanced efficacy of R-1507 in combination with chemotherapy or other targeted agents in several tumors (Gong et al., 2009; Kolb et al., 2010; Wojtalla et al., 2012). In a phase I study of R-1507 in patients with advanced solid tumors R1507 was well tolerated and showed anti-tumor activity in patients with solid neoplasms, in particular Ewing’s sarcoma (Kurzrock et al., 2010). In a phase II study in patients with recurrent or refractory Ewing’s sarcoma, R-1507 induced partial/complete responses in only a subgroup of patients (Pappo et al., 2011). A phase II study of R-1507 in combination with erlotinib was also conducted in advanced-stage NSCLC. The combination did not provide any benefit in comparison to erlotinib alone in unselected NSCLC patients (Ramalingam et al., 2011). Several clinical trials evaluating R-1507, as single agent or in combination with other drugs, are ongoing in patients with various types of solid tumors.

Sch-717454 (19D12; Schering-Plough) is a humanized IgG1 anti-IGF-1R antibody which inhibits IGF binding and autophosphorylation of both IGF-1R/IGF-1R homodimers and IGF-1R/IR heterodimers (but not IR homodimers; Wang et al., 2005). There are no published results from clinical trials with Sch-717454 so far. Four clinical trials have been performed with Sch-717454, one of which is ongoing, one was completed and two terminated.

## SMALL MOLECULE INHIBITORS OF THE IGF-1R TYROSINE KINASE ACTIVITY

**FIGURE 2:**
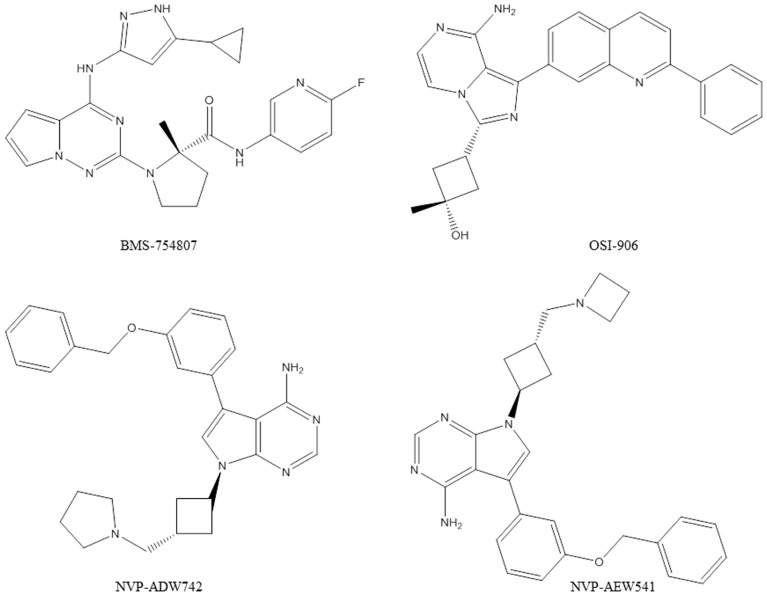
**Chemical structures of the IGF-1R tyrosine kinase inhibitors discussed in this article**.

Several compounds with selectivity toward the IGF-1R tyrosine kinase have entered clinical trials (King and Wong, 2012). Advances in characterization of the structural biology of the insulin and IGF-1R were of great importance for the design of specific IGF-1R inhibitors (De Meyts and Whittaker, 2002). IGF-1R kinase inhibitors are likely candidates to become anti-IGF-1R drugs. However, concerns regarding cross-reactivity with the IR and toxicity, as well as possible mechanisms of resistance still ought to be carefully addressed. A selection of results with small molecule IGF-1R tyrosine kinase inhibitors (**Figure 2**) in pre-clinical models is presented below.

BMS-754807 (Bristol-Myers Squibb) is a potent and reversible inhibitor of the IGF-1R/IR family kinases (Carboni et al., 2009; Wittman et al., 2009). BMS-754807 effectively inhibited the growth of a broad range of human tumor types *in vitro *and was active in multiple xenograft tumor models (Carboni et al., 2009). Combination studies with BMS-754807 revealed synergies when the drug was combined with cytotoxic, hormonal, and targeted agents (Carboni et al., 2009). In triple-negative breast cancers BMS-754807 treatment resulted in tumor regression when combined with docetaxel (Litzenburger et al., 2011). BMS-754807 was tested by the Pediatric Preclinical Testing Program and *in vivo* activity was most commonly observed in the neuroblastoma and rhabdomyosarcoma panels (Kolb et al., 2011). In a subsequent study, a BMS-754807-resistant rhabdomyosarcoma cell line model was developed, which revealed that the platelet-derived growth factor receptor alpha (PDGFRα) plays a role in acquired resistance to BMS-754807. There are now several clinical trials (phase I and II) ongoing with BMS-754807.

NVP-ADW742 and NVP-AEW541 (Novartis) are small molecular weight kinase inhibitors of the IGF-1R, which are specific for the IGF-1R at the cellular level (Garcia-Echeverria et al., 2004; Mitsiades et al., 2004). NVP-ADW742 and NVP-AEW541 have been extensively used in pre-clinical studies in a broad range of human cancer models. However, these compounds were not considered further for clinical development because of toxicity problems observed during the pre-clinical testing phase. The potential of NVP-ADW742 and NVP-AEW541 as single agents or in combination with chemotherapeutic drugs human was investigated in acute myeloid leukemia, Ewing’s sarcoma, medulloblastoma, neuroblastoma, and small cell lung cancer (Scotlandi et al., 2005; Warshamana-Greene et al., 2005; Guerreiro et al., 2006b; Tanno et al., 2006; Doepfner et al., 2007; Tazzari et al., 2007; Urbanska et al., 2007). In atypical teratoid/rhabdoid tumor cells of the central nervous system, NVP-AEW541 was shown to inhibit cell proliferation and survival by blocking IGF-1R and IR activation by autocrine loops involving IGFs and insulin (Arcaro et al., 2007). In colorectal cancer, studies with NVP-AEW541 suggested that a combination therapy targeting both EGFR and IGF-1R could be a promising approach (Kaulfuss et al., 2009). A study in rhabdomyosarcoma also underscored the therapeutic potential of simultaneous targeting of IGF-1R and human epidermal growth factor receptor 2 (HER2) to abrogate resistance (Abraham et al., 2011). In pediatric glioblastoma co-treatment of the PDGFR inhibitor imatinib with NVP-AEW541 resulted in a highly synergistic interaction *in vitro* and increased efficacy *in vivo *(Bielen et al., 2011).

OSI-906 (Astellas Pharma) is a potent, selective, and orally bioavailable dual IGF-1R/IR kinase inhibitor which has demonstrated *in vivo* efficacy in tumor models and is currently in clinical testing (Mulvihill et al., 2009). The activity of OSI-906 in combination with standard chemotherapies was documented in colorectal cancer models (Flanigan et al., 2010). Simultaneous administration of OSI-906 and doxorubicin also significantly enhanced the anti-tumor effect of doxorubicin (Zeng et al., 2012). In human tumor cells co-expressing IGF-1R and IR, it was reported that co-targeting IGF-1R and IR with OSI-906 provides superior anti-tumor efficacy compared with targeting IGF-1R alone using a neutralizing antibody (Buck et al., 2010). Another study described predictive biomarkers for OSI-906 in colorectal cancer (Pitts et al., 2010). Baseline gene expression data from cell lines and xenografts, in combination with IGF-1R detection by *in situ* hybridization and *KRAS* mutational status, was able to accurately predict OSI-906 sensitivity (Pitts et al., 2010). There are now several clinical trials (phase I and II) ongoing with OSI-906.

## CONCLUSION

The available data from the first clinical trials with agents targeting the IGF-1R have been positive enough to launch several phase II and III trials in various human cancers. The IGF-1R antibodies appear to have a favorable safety profile and have been demonstrated to reduce IGF-1R signaling in patients. Concerning the IGF-1R tyrosine kinase inhibitors, the first published data from clinical trials are still awaited. There have been several cases of responses in phase I and II trials with anti-IGF-1R antibodies, but these agents will most likely not be useful in unselected patient populations. In addition, some phase II and III trials have been suspended or terminated, because of lack of efficacy of the antibodies (such as figitumumab in NSCLC). The identification of predictive biomarkers is of crucial importance for the further development of anti-cancer therapies based on anti-IGF-1R agents (King and Wong, 2012). In conclusion, there are multiple challenges still ahead, including the multiplicity of potential cancer indications and drug combinations, as well as the need of biomarkers for resistance and sensitivity.

## Conflict of Interest Statement

The author declares that the research was conducted in the absence of any commercial or financial relationships that could be construed as a potential conflict of interest.
